# Assessment of Gross Motor Skills Performance in Italian Children with and Without Visual Impairment

**DOI:** 10.3390/children12091197

**Published:** 2025-09-08

**Authors:** Giulia Chiara Castiglioni, Giulia Hirn, Marco Lippolis, Matteo Porro

**Affiliations:** 1 Postgraduate School of Physical Therapy and Rehabilitative Medicine, University of Milan, Via Festa del Perdono 7, 20122 Milan, Italy; marco.lippolis@unimi.it; 2 School of Exercise Sciences, University of Milan, Via G. Colombo 71, 20133 Milan, Italy; giuliahirn@gmail.com; 3 Paediatric Physical Medicine & Rehabilitation Service, Neonatal Intensive Care Unit, Fondazione IRCCS Ca’ Granda Ospedale Maggiore Policlinico, Via Manfredo Fanti 6, 20122 Milan, Italy; matteo.porro@policlinico.mi.it

**Keywords:** visual impairment, children, blindness, gross motor skills, motor development, TGMD-2

## Abstract

**Highlights:**

**What are the main findings?**

- First Italian study assessing gross motor skills in children with visual impairment using the TGMD-2, showing significantly lower performance compared to sighted peers.
- The gross motor skill age of children with visual impairment is markedly lower—by about 4–5 years—than their chronological age.

**What is the implication of the main finding?**

- Provides a benchmark for international comparison and underscores the need for early, targeted educational and rehabilitative programs to enhance motor competence.
- Highlights that children with visual impairment may face significant challenges in participating in motor play and sports with same-age peers, impacting inclusion.

**Abstract:**

Background/Objectives: Vision plays a key role in acquiring and automating fundamental movement skills. Evidence from Italy is scarce. This study compared Test of Gross Motor Development–2 (TGMD-2) performance between Italian children with visual impairment (VI) and sighted peers and explored differences by degree of VI (severe VI vs. blindness). Methods: This was a cross-sectional study including 38 children (VI: *n* = 19, 5–12 y; sighted: *n* = 19, 5–11 y). VI was classified per the WHO criteria. TGMD-2 was administered with adaptations suitable for VI (e.g., high-contrast cones, auditory balls, verbal cueing). Group differences (VI vs. sighted; blindness vs. severe VI; severe VI vs. sighted) were analyzed with Mann–Whitney U (α = 0.05). For participants > 10 y, raw scores were analyzed and age-equivalent scores were summarized to calculate developmental gaps. Results: Children with VI scored lower than sighted peers on locomotor, object control, and total TGMD-2 scores (all *p* < 0.001). Within VI, blindness was associated with lower locomotor, object control, and total scores than severe VI (*p* = 0.013; *p* = 0.043; *p* = 0.013). Children with severe VI also scored lower than sighted peers across outcomes (all *p* < 0.001). Based on age-equivalent estimates, average gross motor performance in VI was ~4–5 years below chronological age; values < 3 years were set to 3 years for calculation (the floor effect). Conclusions: Italian children with VI show reduced gross motor competence compared with sighted peers, with a substantial developmental lag. Findings support early adapted physical education and structured movement opportunities to promote participation and inclusion. Future studies should examine long-term outcomes and contextual factors shaping motor competence in youth with VI.

## 1. Introduction

Sight is the primary channel of interaction with the environment. It guides and regulates the acquisition, differentiation, and automation of motor skills. Visual information plays a significant role in motivating children to move by providing details regarding the distance and direction of movements and objects, assessing potentially hazardous situations, and calibrating movements. In addition, observing others prompts children to learn movements [1]. Impaired visual function during early development significantly affects the progression of motor abilities and acquisition of skills. From early infancy, children with visual impairment (VI) may experience delays in achieving important motor milestones such as controlling their head movements [2]. These children perform tasks such as sitting, crawling, standing, and walking later than children with sight [3,4,5,6]. Moreover, as children mature, they may encounter challenges related to spatial orientation, temporal coordination, perceiving sensory information, developing body awareness, acquiring self-care skills, and adjusting their posture [7,8,9,10]. VI also negatively affects gross motor skills; however, studies that analyze this topic are limited in number due to a lack of validated tools for children with VI [7]. Gross motor competence is defined as proficiency in a range of fundamental movement skills (such as running, throwing, catching, and jumping) that are ideally acquired during preschool and primary school years [11] and play a significant role in development and providing opportunities for a healthy and active life [12,13,14,15]. Bakke et al. [7] concluded that the only tests that presented valid and reliable data in studying the motor skills of children with VI were the Test of Gross Motor Development-2 (TGMD-2; which tests gross motor skills) and Movement ABC-2 (which studies manual dexterity, balance, and object control). On considering only gross motor skills and excluding praxic–manipulative abilities, the most reliable test for assessing these skills is TGMD-2, which is now available in its third version (TGMD-3) that was not evaluated by Bakke et al. [7].

According to Bakke’s systematic review, TGMD was used in five studies [16,17,18,19,20], all of which were deemed of high quality and reliability based on the Critical Review Form-Quantitative Studies [21]. The first study to use the TGMD-2 to investigate motor skills in children with VI was conducted by Houwen et al. in the Netherlands [19]. More recently, within the Camp Abilities sports program (United States), quantitative studies applied TGMD-2/3 to examine case–control differences, correlates (age, sex, degree of VI), three-year locomotor trajectories, links with BMI/fitness/physical activity, relations with perceived motor competence, and initial psychometric properties of the Total Body Developmental Sequences [17,18,22,23,24,25,26,27].

These studies primarily examined children with VI in the United States and the Netherlands, confirming that validated tools for assessing gross motor skills in this population, such as the TGMD-2 and TGMD-3, are used in limited capacities worldwide.

Assessing gross motor skills in children is important because reduced motor performance can have long-term consequences [28,29]. Winnick reported [30] that adolescents with VI may have difficulty performing activities involving gross motor skills. Poor gross motor performance could affect the social participation of children with VI, considering that gross motor skills serve as the foundational elements for acquiring more intricate movements that are essential for engaging in sports and games and actively participating in physical activities [31]. Brian et al. [23] documented in their longitudinal study that motor difficulties in children with VI do not always manifest as a developmental delay but can progress into “arrested development,” where motor skill acquisition stagnates and may persist over time. Some studies have shown that targeted activity programs can optimize motor performance in children with VI [4,32,33,34,35,36]. A relevant example is Camp Abilities, an international sports education program for children and youth with VI, which provides substantial benefits in motor development and self-efficacy among participants [27]. Thus, gaining a comprehensive understanding of the qualitative aspects of motor skill performance in children with VI is important because it yields valuable insights into designing early intervention strategies aimed at reducing the enduring effects of poor motor skills in children with VI. Some studies [13,32,37,38] have suggested that environmental opportunities and movement barriers, rather than VI, may influence motor skill performance in individuals with VI; hence, further investigation is necessary to understand how these factors manifest in different sociocultural contexts. Most available data on this topic is from the United States and the Netherlands [7,27], highlighting the need for country-specific research. To address this gap, this study aimed to analyze the gross motor skills of Italian children with VI and a sample of peers without VI using the TGMD-2 test. Additionally, the collected data were compared with age-normative values from the TGMD manual and previous studies conducted in the United States and the Netherlands.

To date, similar studies have not been conducted in Italy. The diagnosis of VI in Italian children is usually included in early rehabilitation programs. Special schools for children with VI do not exist, and children are integrated into general education classrooms with children without VI. Physical education at school is usually limited to 2 h per week. The extracurricular sports activities available to children with VI are limited. Inspired by the growing number of studies on gross motor skill performance in children with VI, this study was designed to expand knowledge in this field by adding Italian data to the existing international body of research. The collected data can serve as a valuable reference for comparisons and provide an empirical foundation for the enhancement of early intervention programs for Italian children with VI.

## 2. Materials and Methods

### 2.1. Participants

A total of 38 Italian children were recruited. 19 children with VI (age, 5–12 years; mean age 9.3 years or 111.11 months), comprising 14 boys and 5 girls, were included. They were recruited from a summer camp organized in Tirrenia (Italy) by a nonprofit Italian sports association (Real Eyes Sport ASD). Only children without other disabilities (except VI) were selected based on their medical records.

According to the definition of the World Health Organization (WHO) based on the International Classification of Diseases 11 (2018), children were classified as having VI as follows: 10 children had severe VI (visual acuity worse than 20/200 but equal to or better than 20/400). 1 child had central visual field restriction. 9 children had blindness (visual acuity worse than 20/400). 18 children had congenital diseases and were visually impaired since birth. 1 child had normal sight up to the age of 6 years and then developed blindness because of a cerebral tumor.

Of the 19 children with VI, one each had agenesis of the anterior optic pathways and microphthalmia; retinal detachment in the hyperplastic primary vitreous; microphthalmus and nystagmus; bilateral hyperplastic primary vitreous; Norrie disease; optic pathway glioma undergoing chemotherapy for neurofibromatosis type 1; congenital glaucoma; retinopathy of prematurity; osteopetrosis; microphthalmia; Peter’s anomaly; and pseudocoloboma of the macula and Leber congenital amaurosis; 2 had Leber congenital amaurosis; and 4 had albinism.

19 children with an age appropriate to their grade level were recruited from a leisure center in northern Italy (age, 5–11 years; mean age, 8.2 years or 98.53 months). 13 were boys and 6 were girls.

Informed consent was provided by the children’s parents, and verbal consent was obtained from the children. The procedures were performed in accordance with the ethical standards of the Faculty of Medical Sciences at the University of Milan. Review and/or approval by an ethics committee was not required for this study because it was an observational study in which no treatment was administered to the participants.

### 2.2. Instruments

The TGMD-2 was selected to assess the gross motor performance of the participants as it is a widely adopted instrument for evaluating primary school children [39]. Houwen et al. [20] concluded that it is an appropriate tool for assessing gross motor performance in primary school children with VI. It has been validated for children of 3–10 years of age, but has been used for children older than 10 years [19].

The second version of the test (TGMD-2) was utilized instead of the more recent TGMD-3 [40] because, at the time of data collection in 2020, the TGMD-2 was adopted in most studies, especially those that comprehended children with VI. This guarantees an easier comparison with the results obtained in other countries.

TGMD-2 consists of two subtests that assess locomotor (LM) and object control (OC) abilities. Each subtest measures six gross motor skills. The LM subtest assesses the skills involved in moving the center of gravity from one point to another: running, hopping, leaping, horizontal jumping, and sliding. The OC subtest measures skills involved in throwing and receiving objects: striking a stationary ball, stationary dribbling, catching, kicking, overhead throwing, and underhand rolling.

Two trials were performed for each gross motor skill, and both contributed to the final score. The examiner measured the quality of the movement using the performance criteria provided by the test manual, which assigned 0 points if the criterion was absent and 1 point if present. For example, for running skills, four criteria have to be evaluated for each trial, for a total maximum score of 8 points: moving in opposition to legs with elbow bent, a brief period where both feet are off the ground, foot placement landing on heel or toe (not flat-footed), and non-support leg bent approximately 90°.

The conversion tables provide raw scores to be transformed into standard scores (raw scores standardized by age), percentiles, and age equivalencies. The gross motor quotient and descriptive ratings were derived from a standard score. Descriptive ratings classify a child’s age-standardized performance into the following categories: very poor, poor, below average, average, above average, superior, and very superior, with average representing the normative score for a given age group.

In the present study, raw scores were used in the analyses of children older than 10 years (*N* = 5) as this age group falls outside the range of TGMD-2 normative data. For these participants, calculation of standard scores, gross motor scores, or descriptive ratings was not possible; however, the conversion of raw scores into age equivalencies was still feasible.

**Figure A1:**
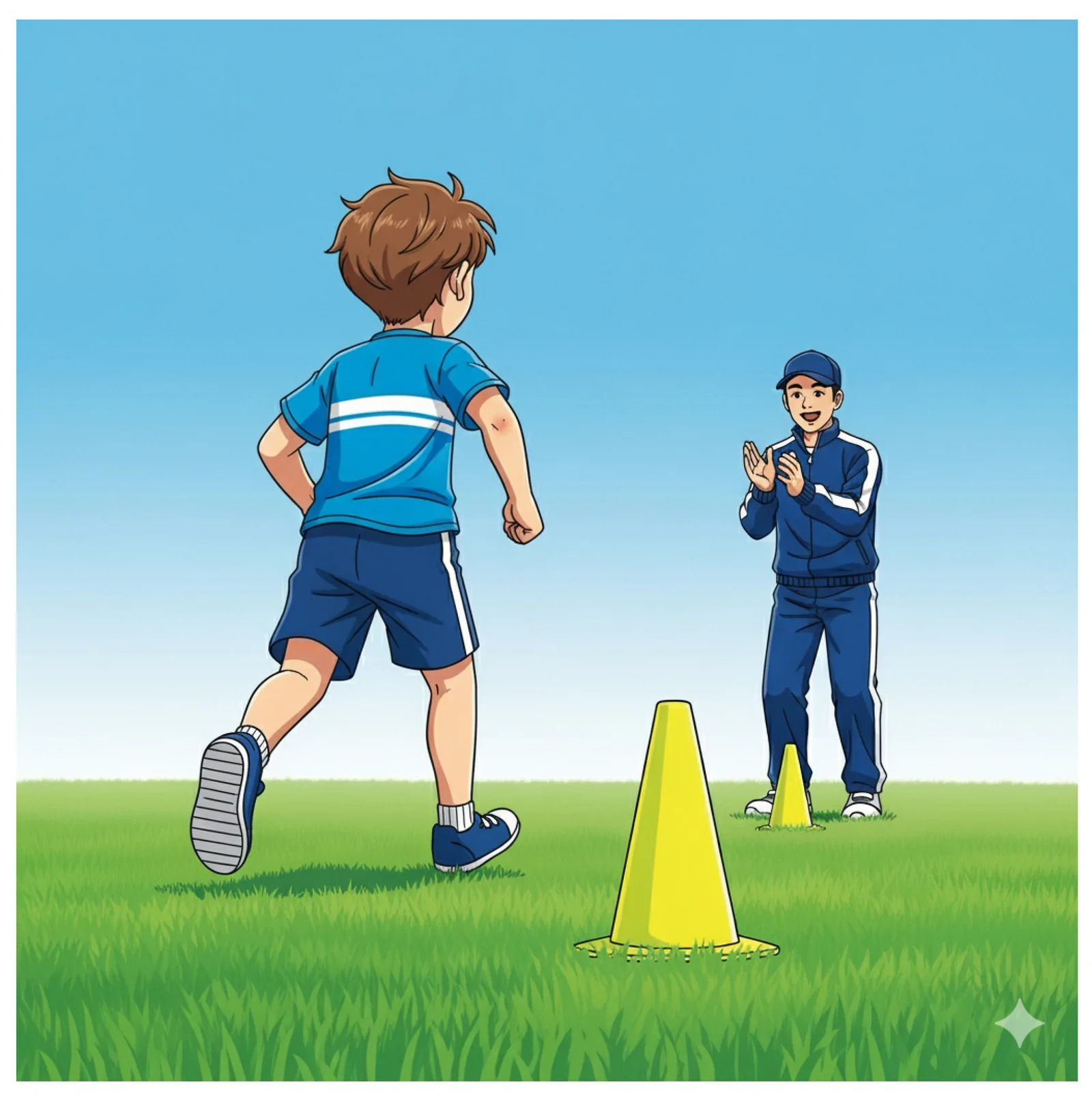
Locomotor tasks—auditory and high-contrast visual guidance. The child moves from the start to the stop line, both marked by high-contrast cones placed to maximize residual vision. The examiner provides continuous auditory cueing (e.g., clapping or calling) to indicate direction and endpoint. These adaptations were applied across all TGMD-2 locomotor tasks (run, gallop, hop, leap, horizontal jump, slide). Illustration generated with Google Gemini (text-to-image) under author supervision; for explanatory purposes only and not depicting study participants.

**Figure A2:**
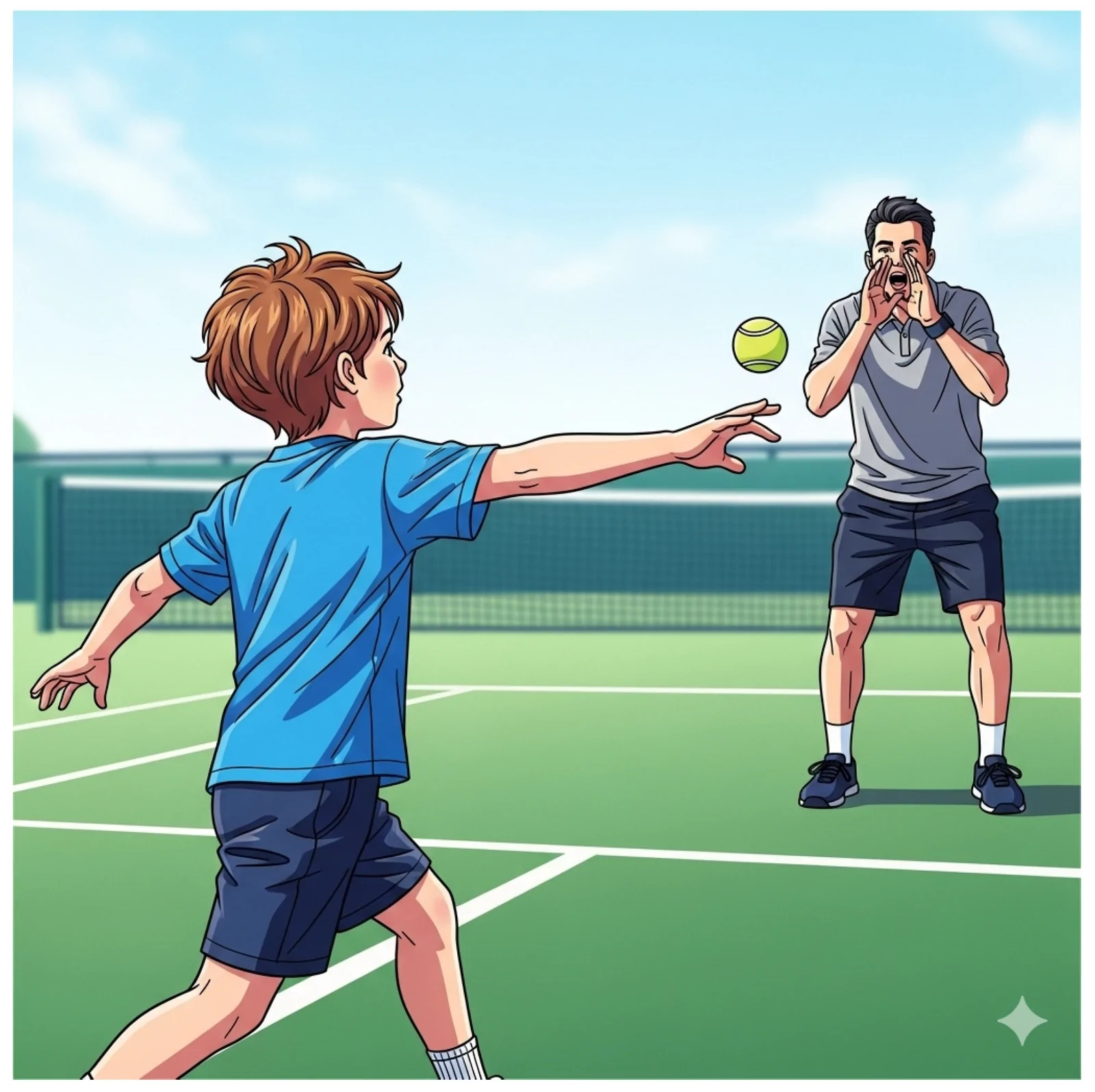
Object control subtests—throwing toward an acoustic/voice target. The child throws the ball toward the operator who provides a continuous voice cue (or clapping); this guidance strategy is applicable to TGMD-2 object control items that require a throw or a kick. Illustration generated with Google Gemini (text-to-image) under author supervision; for explanatory purposes only and not depicting study participants.

**Figure A3:**
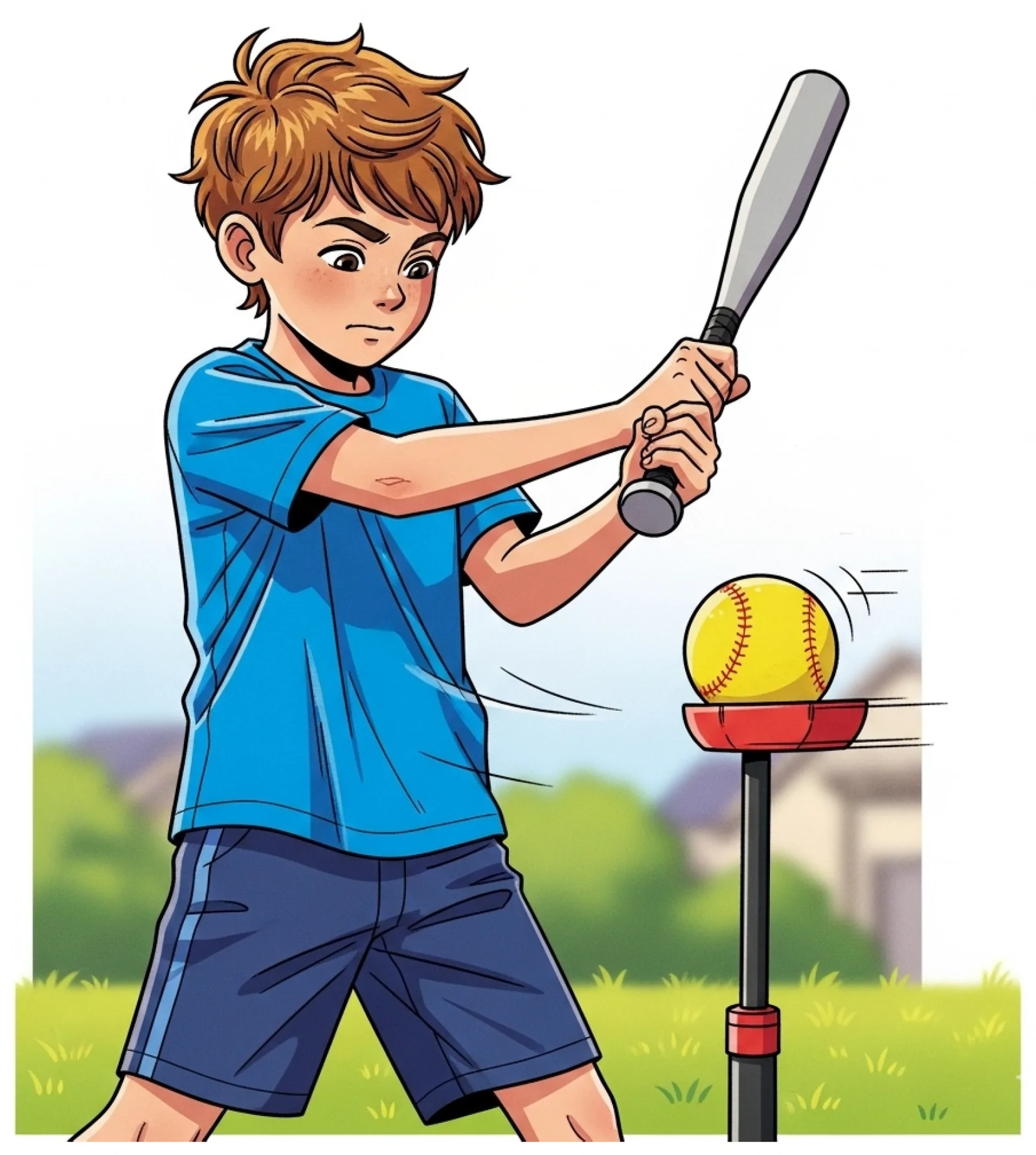
Striking a stationary ball (TGMD-2)—tactile preview and sound ball. The ball is positioned on a batting tee; before each attempt, the child is allowed to tactually explore the tee and ball to locate them in space. A sound-emitting ball is used to assist children without residual vision. Illustration generated with Google Gemini (text-to-image) under author supervision; for explanatory purposes only and not depicting study participants.

The original test was adapted to enable children with VI to perform the test, using adjustments mostly based on Howen [19] and Brian et al.’s studies [41]: big, bright, colored cones were used to mark the start and the end of the lane to indicate child position; the children were allowed to test the items before the test was administered; owing to the impossibility of visually explaining the exercises to children with blindness, the examiner was allowed to let the child perceive the required movement; vocal feedback to maintain direction during the locomotor exercises was given by the examiner; and bright acoustic balls were used for the OC tests. Table 1 provides a detailed explanation of the modifications applied to the equipment and original TGMD-2 test procedure. Visual examples of the VIspecific TGMD2 adaptations (highcontrast cones, auditory balls, examiner verbal cueing) are provided in Appendix A (Figure A1, Figure A2 and Figure A3).

### 2.3. Procedure

Both groups of children with and without VI were assessed using the TGMD-2 test during the summer of 2020. The children without VI were tested on the playground of a local leisure center. Children with VI were tested in a sports field during the summer camp activities that they attended. Each child was tested individually by the same examiner, a sports science student, and a track-and-field instructor, under the supervision of a kinesiologist. All test sessions were video recorded to allow for a more accurate scoring process, which could be conducted at a later stage after test administration.

### 2.4. Data Analysis

All statistical analyses were performed using the IBM SPSS Statistics (version 28, IBM Corp., Armonk, NY, USA). Descriptive statistics were calculated for children with and without VI.

The dependent variables were LM and OC subset raw scores and the total raw scores on the TGMD-2. Shapiro–Wilk test was performed to evaluate the distribution of these variables, which showed non-normal distribution of variables. Owing to the results of the Shapiro–Wilk test and the low number of children with VI, the non-parametric Mann–Whitney U test was used to compare the variables between children with and without VI. An alpha error < 0.05 was considered as significant.

Children with VI were divided into two subgroups according to the degree of deficits: those with blindness and those with severe VI. Differences in the dependent variables were tested between the groups of children with severe VI and children without VI using the Mann–Whitney U test.

The dependent variables for children with VI and both subgroups, severe VI and blindness, were compared with the results reported by Howen et al. [19], Wagner et al. [17], and Haibach et al. [18], using the Wilcoxon rank-sum test.

## 3. Results

### 3.1. Gross Motor Skills of Children with and Without VI

**Figure 1:**
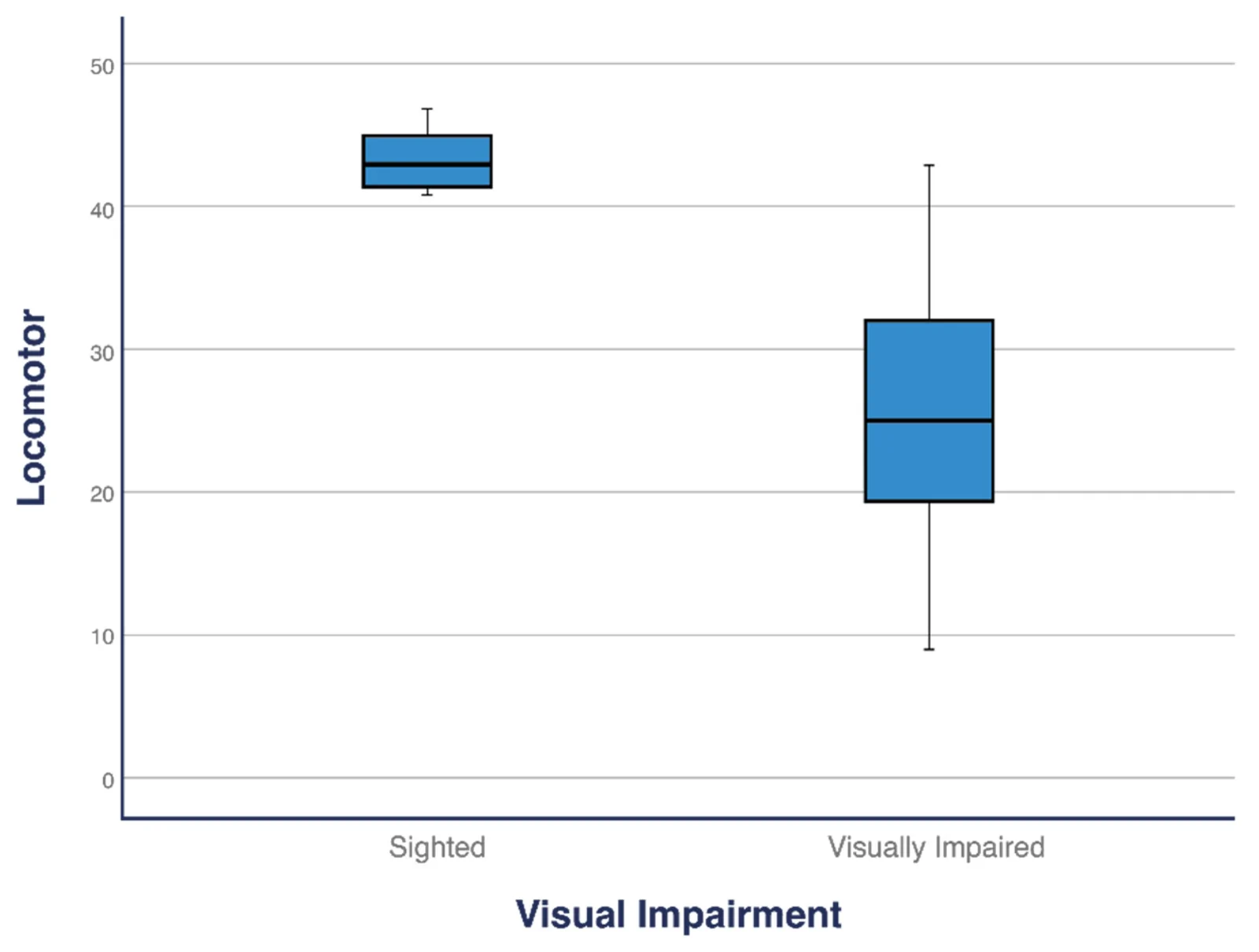
Box plot comparison of locomotor scores between children with and without VI. Key result: the median locomotor score was lower in children with VI (25) than in sighted children (43); group difference *p* < 0.001 (see Table 2).

**Figure 2:**
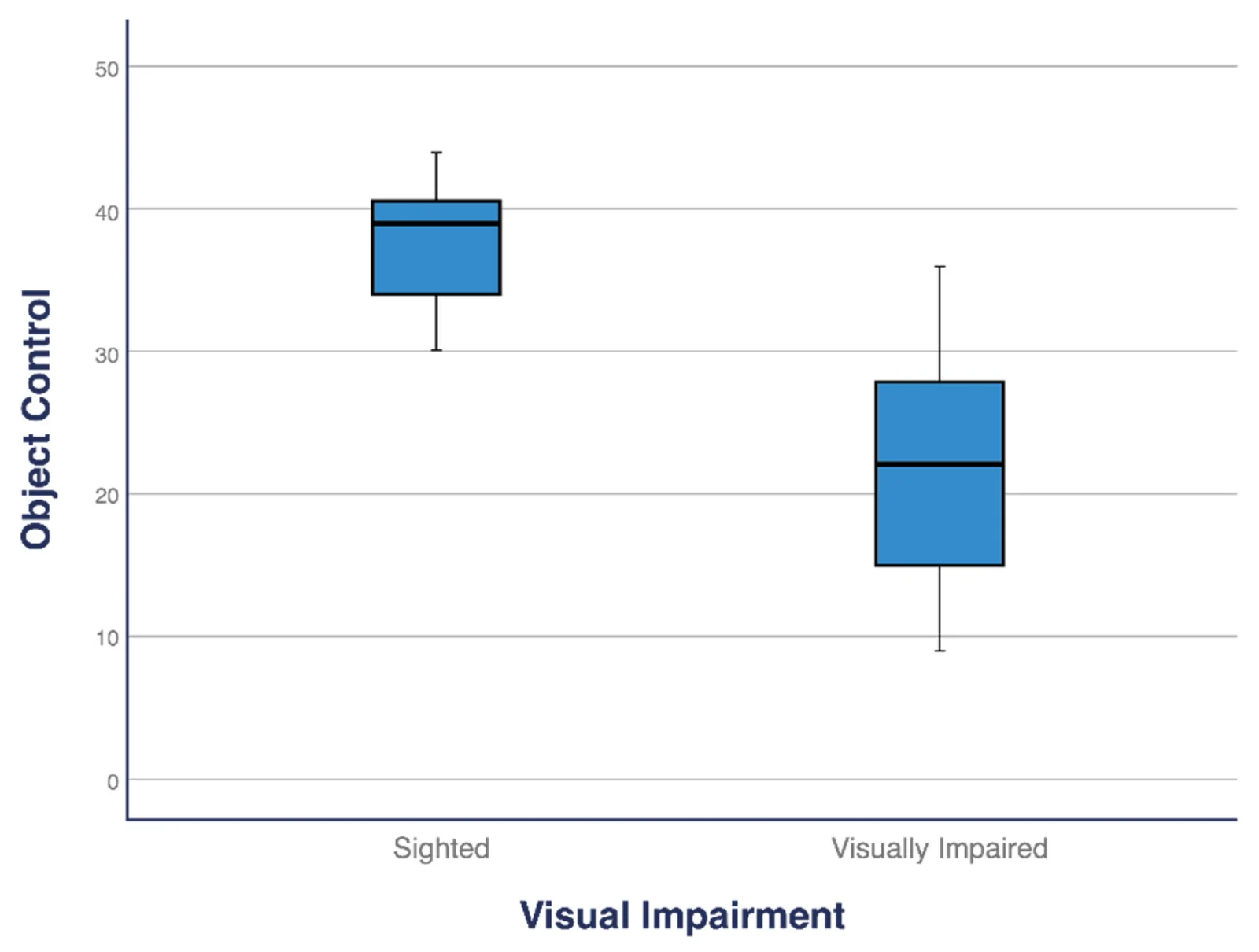
Box plot comparison of object control scores between children with and without VI. Key result: the median object control score was lower in children with VI (22) than in sighted children (39); group difference *p* < 0.001 (see Table 2).

**Figure 3:**
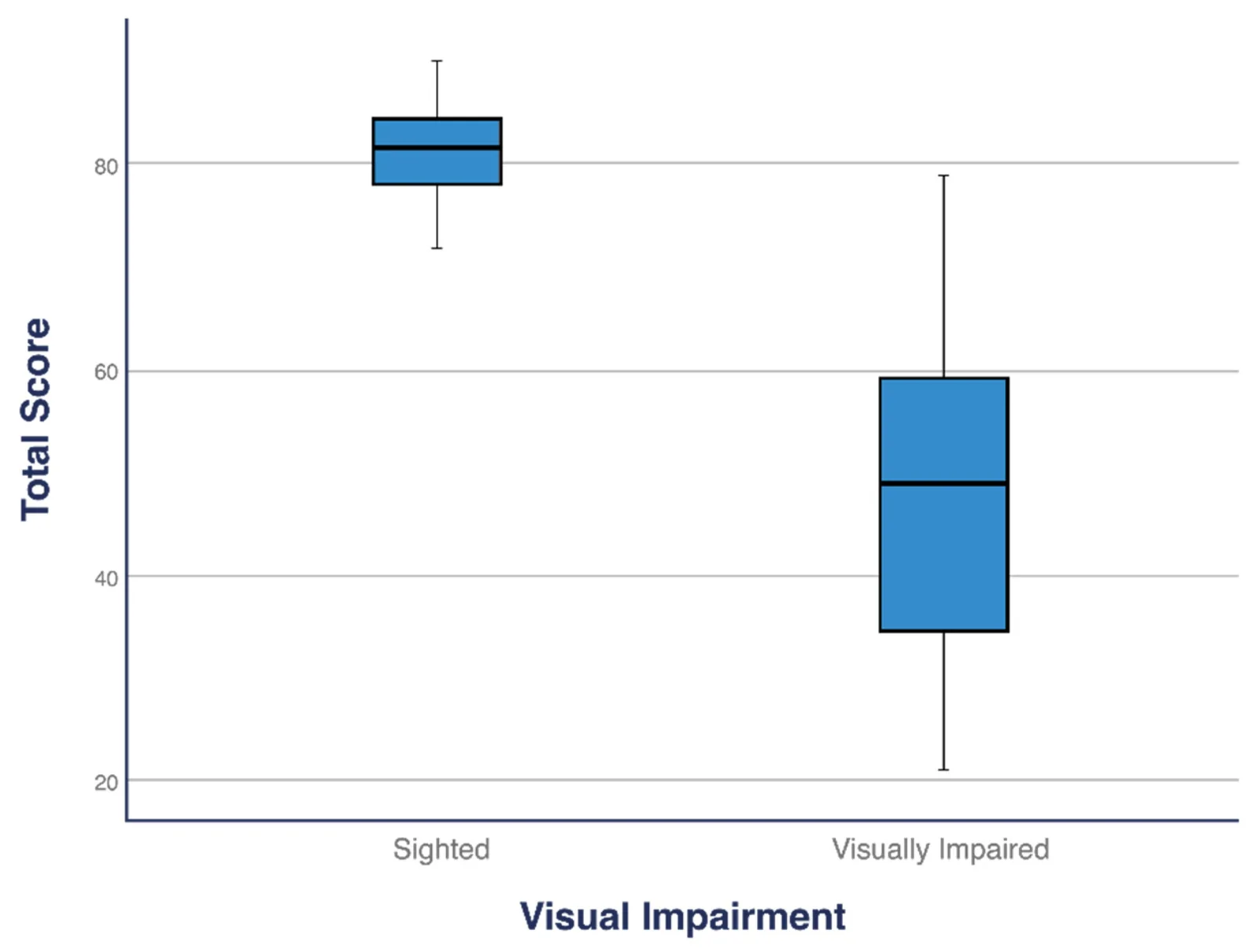
Box plot comparison of total scores between children with and without VI. Key result: the median total score was lower in children with VI (49) than in children without VI (82); group difference *p* < 0.001 (see Table 2).

Children with VI differed from those without VI in both the subtests (LM and OC) and total score. The total score of children with VI (*M* = 47.63) was significantly lower than that of children without VI (M = 78.42; *p* < 0.001). Similarly, children with VI scored significantly lower in both the LM (M = 25.47) and OC (M = 22.16) subtests than children without VI (LM: M = 42.26; OC: M = 36.16 and LM: *p* < 0.001, OC: *p* < 0.001; Table 2). The median total score, LM, and OC for children with VI were significantly lower than those for children without VI (Figure 1, Figure 2 and Figure 3).

### 3.2. Association of Gross Motor Skills Performance and Degree of VI

Children with different degrees of VI were recruited in our study group; hence, two subgroups were compared: children with blindness (age 111.0 months, standard deviation [SD] 27.16) and children with VI (age 111.20 months, SD 24.9). Children with blindness scored significantly lower than children with severe VI in both the LM (*p* < 0.013) and OC (*p* < 0.043) subtests and in the total score (*p* < 0.013; Table 3).

### 3.3. Gross Motor Skills of Children with Severe VI Compared to Children Without VI

A comparison of the mean gross motor performance between the children with blindness and those with severe VI revealed a substantial difference (Table 3). This finding led to a further comparison between the scores of children without VI and those of children with severe VI. Children with severe VI scored significantly lower than those without VI in the LM (*p* < 0.001), OC (*p* = 0.001), and total score tests (*p* < 0.001; Table 4).

### 3.4. Gross Motor Skills of Italian Children with Severe VI Compared to Dutch Children with Severe VI

Among the few studies that used the TGMD-2 to analyze gross motor skills in children with VI, Houwen et al.’s study [19] was identified as the most suitable for comparison. This study provided the mean, median, and SD of the subtest and total raw scores for Dutch children with VI within a similar age range (6–11 years, mean age, 9.20 years) as the participants in the present study. Moreover, similar TGMD-2 adaptations were applied in both studies to enable children with VI to complete the test trials.

Houwen et al. [19] divided the sample into two subgroups: children with moderate VI (*N*= 7) and children with severe VI (*N* = 7), according to the WHO definition; no children with blindness were included in the study. Italian children with severe VI and Dutch children with severe VI were compared based on this classification.

Italian children scored significantly lower than Dutch children in the LM subtest (*p* = 0.015) and total score (*p* = 0.02), whereas no significant difference was found in the OC subtest (*p* = 0.10; Table 5).

### 3.5. Gross Motor Skills of Italian Children with VI Compared to US Children with VI

Most studies analyzing gross motor skills in children with VI originated in the US; hence, we considered it relevant to compare the scores obtained by an Italian sample of children with VI with those of a group of American children with VI.

However, upon reviewing the US studies to extract comparable data, we found that most of them adopted a classification system for VI that differed from the WHO classification used in this study. Many American studies rely on the United States Association for Blind Athletes classification system, which includes three categories:

- B3: Best corrected vision between 20/200 and 20/599, legally blind.
- B2: Best corrected vision between 20/600 and beyond, travel vision.
- B1: Totally blind.

In contrast, the WHO classification defines individuals with visual acuity worse than 20/200 but equal to or better than 20/400 as having severe VI, whereas those with visual acuity worse than 20/400 are classified as blind.

Therefore, we selected the study by Wagner et al. [17] for comparison, as it specifically examined children classified as blind according to the WHO system, allowing for direct comparison with children with similar VI assessed in the present study.

Italian children with severe VI were compared to the B3 category in the study by Haibach et al. [18], which was chosen because of its large sample size and clear reporting of LM and OC subtest scores. Although the B3 category and the WHO-defined severe VI category were not entirely equivalent, we considered it appropriate to proceed with the comparison.

Italian children with severe VI scored significantly lower than American children in the B3 category in the OC subtest (*p* = 0.007) and total (*p* = 0.037) scores. However, no statistically significant differences were found in the LM subtest scores (*p* = 0.72; Table 6).

The comparison between the scores of Italian and American children with blindness did not reveal any statistically significant differences in either the LM (*p* = 0.72) or OC subtests (*p* = 0.34; Table 7).

### 3.6. Gross Motor Skill Performance of Italian Children with VI Compared with TGMD-2 Normative Data

Table 8 presents the age-equivalent values, gross motor score, and descriptive ratings, which describe the global gross motor performance of each child with VI of 3–10 years of age. Raw scores and descriptive ratings for each subtest (LM and OC) are reported.

For five participants, calculation of the gross motor quotient and descriptive ratings was not possible, as they exceeded the age limits of 10 years and 11 months. However, calculation of the age equivalent for these participants based on their raw scores was possible and considered appropriate and relevant.

**Figure 4:**
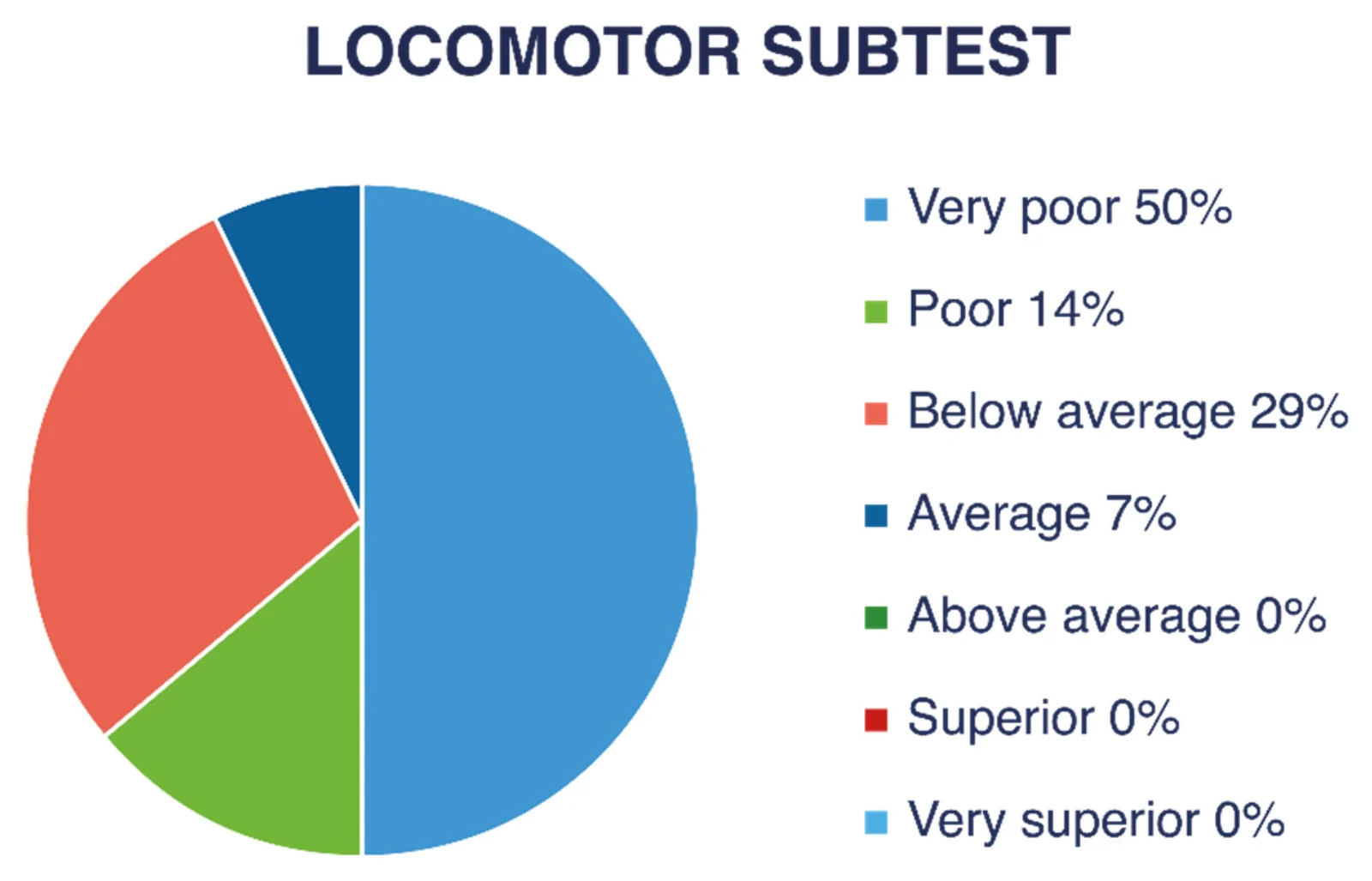
Descriptive ratings of locomotor subtest for children with visual impairment. Key result: in the VI group, 50% were rated ‘very poor’, 14% ‘poor’, 29% ‘below average’, and 7% ‘average’; none were ‘above average’ or higher.

**Figure 5:**
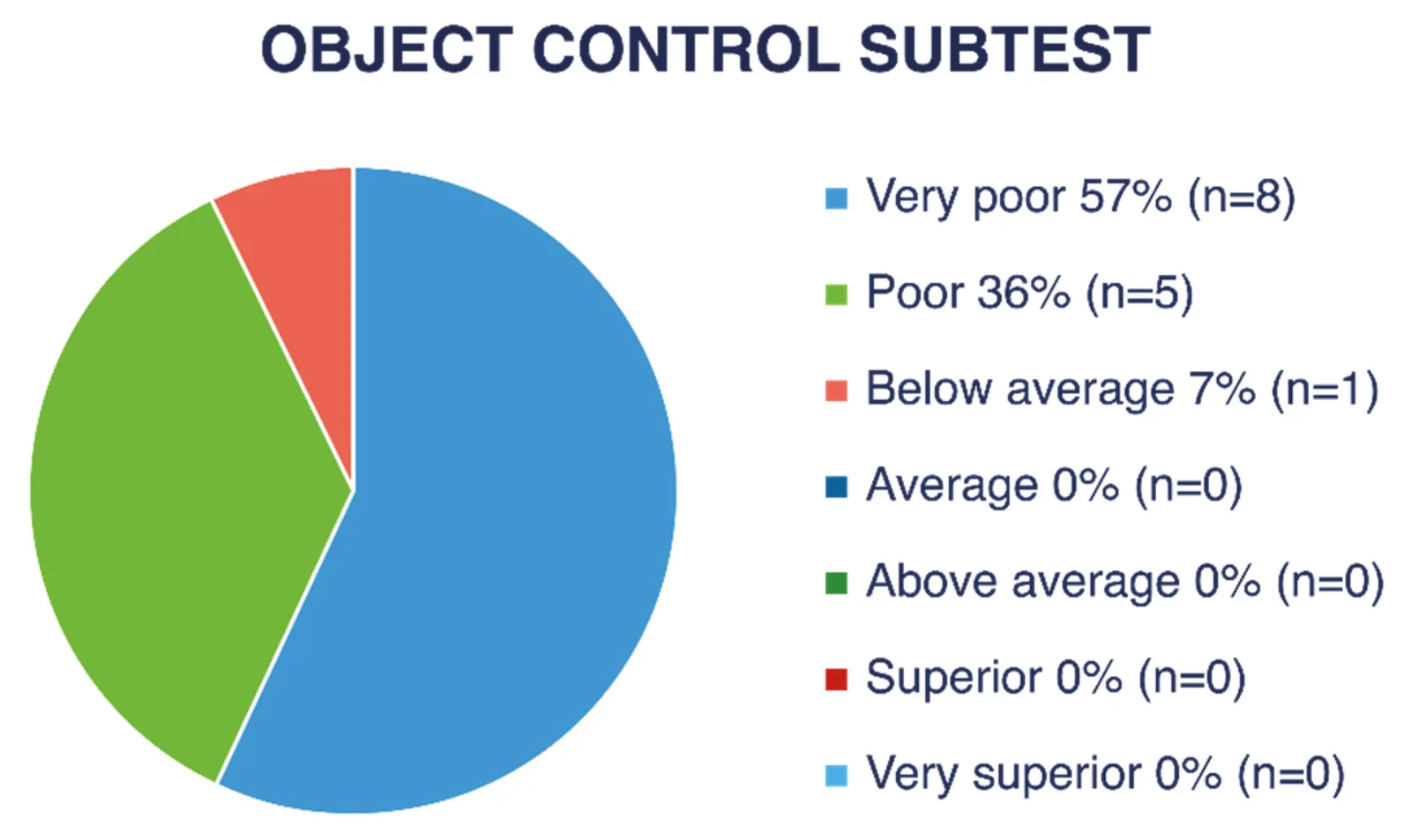
Descriptive ratings of object control subtest for children with visual impairment. Key result: in the VI group, 57% (*n* = 8) were ‘very poor’, 36% (*n* = 5) ‘poor’, 7% (*n* = 1) ‘below average’; no children were ‘average’ or above.

**Figure 6:**
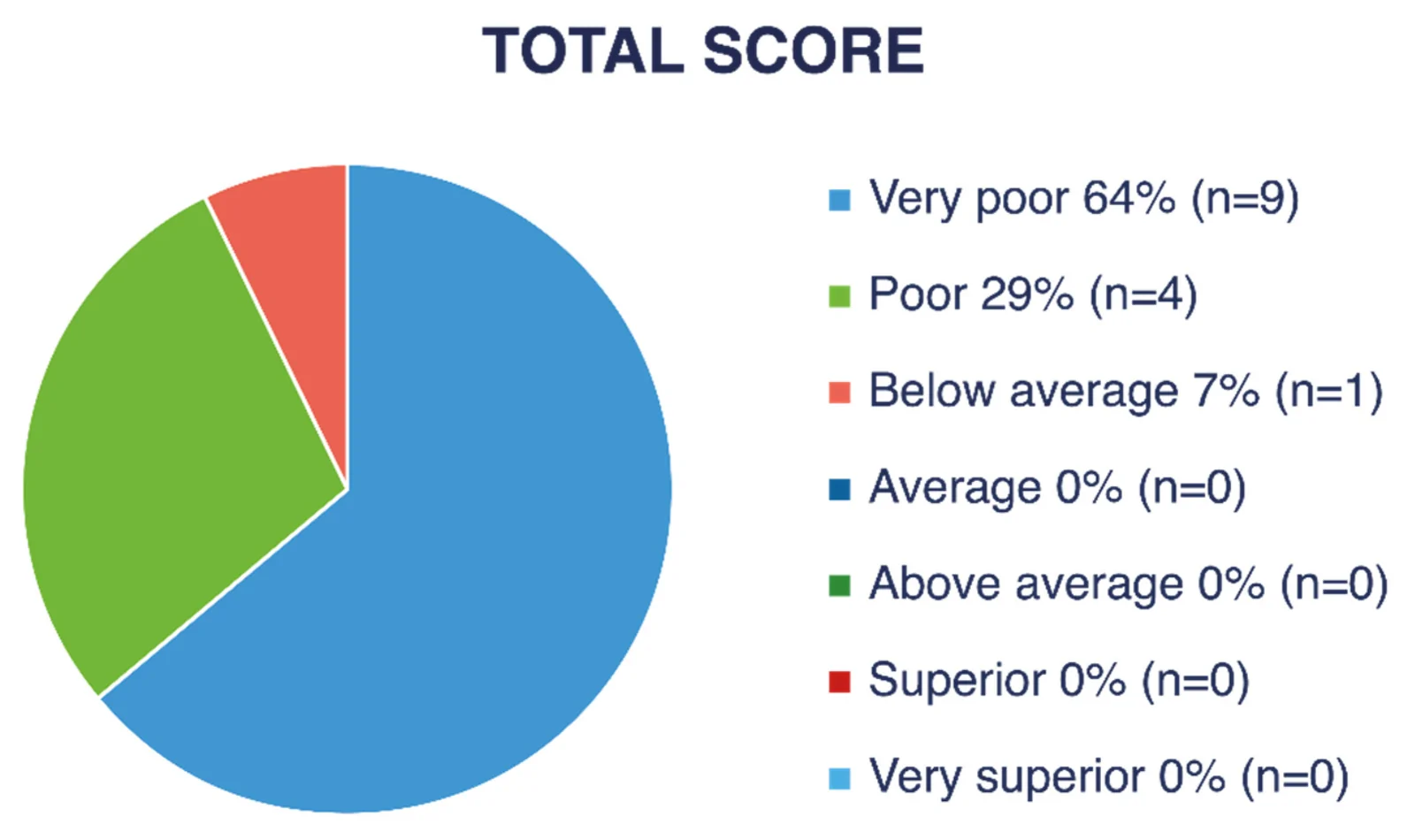
Descriptive ratings of total score for children with visual impairment. Key result: 64% (*n* = 9) were ‘very poor’, 29% (*n* = 4) ‘poor’, and 7% (*n* = 1) ‘below average’ on the global score.

Figure 4 and Figure 5 show descriptive ratings for each subtest (LM and OC). As shown in Figure 6, nine children (47% of the children with VI) obtained a “very poor” descriptive rating in the global score, four children (21%) received “poor,” and one child (5%) received a “below average” rating.

A “very poor” rating corresponds to a gross motor quotient below 70, a value found in only 2.34% of the normative population without VI. The calculated average gross motor quotient was approximately 61.5, which corresponds to a percentile below 1 in the normative American population without VI.

By rounding the performance of children who obtained an age-equivalent score of <3 years to 3 years and 0 months, the gross motor performance age of children with VI was, on average, 58 months (4 years and 10 months) lower than their chronological age in LM skills and at least 59 months (4 years and 11 months) lower in OC skills based on estimations.

### 3.7. Gross Motor Skill Performance of Italian Children Without VI Compared with TGMD-2 Normative Data

The normative data of the TGMD-2 are based on a population of children without VI from the US; hence, it was considered useful to compare the scores obtained by Italian children without VI with these normative values to acknowledge the potential cultural and demographic differences between Italian and American children without VI.

Of the 19 Italian children without VI, calculation of the gross motor quotient and descriptive ratings was not possible for two as they exceeded the age limit. On analyzing the scores of the remaining 17 children, 58.8% scored within the “average” range, 5.9% in “above average” category, and 35.3% in “below average.” These findings are consistent with those of a recent study that conducted a comprehensive psychometric assessment of the TGMD (in its third version) on a large sample of Italian preschool and primary school children (*N* = 5210; mean age = 8.38 years; SD *=* 1.97). The results demonstrated strong construct validity and reliability of the TGMD-3 for measuring gross motor skills in Italian children across sex and age groups.

## 4. Discussion

This is the first study to investigate gross motor skill performance using the TGMD-2 in Italian school children with and without VI. The findings suggested that Italian children with VI exhibited significantly lower gross motor skills than the Italian children without VI of the same age, as well as compared to normative data from the TGMD-2, which reflects the American normative population without VI [39]. This finding was consistent with those of previous studies that have highlighted the significant role of vision in gross motor skill acquisition [1,2,3,4,5,6]. However, further investigation and quantification of this gap are necessary. Differences in motor performance between children with and without VI were observed for both LM and OC skills. This finding partially contradicted that of a previous study [19] conducted on Dutch children, which reported differences between children with and without VI in OC abilities but not in LM skills. Houwen et al. [19] suggested that the strong LM performance observed in children with VI in their study could be attributed to the frequent engagement in LM activities during daily play and sports participation. In contrast, despite having comparable visual deficits, the participants in the present study exhibited significant difficulties even in the LM subtest, suggesting that these movements may not be practiced as frequently in their daily lives. However, the daily physical activity levels of Italian children were not investigated in the present study. Regarding OC skills, children with VI could have shown worse performance because of the critical role of vision in providing feedback on object position and movement [19,30]. Additionally, controlling objects such as balls, whether using the hands (such as throwing tasks) or feet (such as kicking tasks), is a common and fundamental motor activity in the play routines of children without VI. Therefore, the lack of proficiency in these skills may have detrimental implications for social inclusion, as children with VI may struggle to fully participate in peer group activities involving OC tasks.

The degree of visual acuity was found to influence motor performance in both the LM and OC subtests, and children with blindness had a lower gross motor skill level than children with severe VI, which can be expected, considering the possibility for those with severe VI to rely partially on the visual channel [19]. This result was consistent with those of previous studies, such as those by Haibach et al. [18] and Stribing et al. [25], reinforcing the existing evidence on the impact of VI on motor development. Even with better visual acuity, children with severe VI were unable to achieve motor performance comparable to that of their peers without VI, further underscoring the critical role of visual deficits on development of gross motor skills.

Age-equivalent calculations, percentiles, and gross motor quotients provided by the TGMD-2 test were used to quantify developmental gaps in motor skills between children with and without VI. The results indicated that the average level of motor ability recorded in the group of children with VI was less than 1% of the normative population without VI. Within the limitations of the assessment tool, the motor gap between the two groups could be quantified as over four years on average.

Although the motor milestones in children with VI are achieved later than in their peers without VI [3,4,5,6], whether this gap can be fully closed if it is prominent remains unclear. This concern is particularly relevant, given that the critical period for the acquisition of gross motor skills is during the early years of life [42].

The calculation of age equivalence (Table 6) demonstrated that even children with VI over the age of 10 years exhibited a gross motor age that is several years below their chronological age compared with their peers without VI (on whom the normative data are based). A 11-year-old child with a gross motor age equivalent to that of a 5-year-old child would face significant challenges in engaging in motor play activities with peers of the same age. Moreover, improving motor skills at the age of 11 years is likely to be more difficult than implementing interventions in the early years of life.

These findings were consistent with those of Brian et al. [22], which suggested that delays in motor milestone acquisition in children with VI can progress to a state of stagnation. In that study, a longitudinal analysis of LM skill development over a three-year period conducted using the TGMD-3 showed that participants demonstrated minimal-to-no improvement in gross motor skills, stabilizing at a score plateau of 27–29. This corresponds to a developmental delay of approximately 6 years compared with the normative data. This phenomenon underscores the necessity for targeted educational and rehabilitative interventions to help children with VI overcome the proficiency barriers in motor competence.

Comparing the data obtained in the present study with those collected in other countries, such as the studies by Howen et al. [19] and Haibach et al. [18], significant room for improvement exists for these children: Dutch children with similar VI scored significantly higher than Italian children in LM skills, while American children with similar VI showed significantly higher scores in OC ability. Despite the limited sample size in both studies, increasing motor activity in children with VI during the early years of life may lead to motor skills comparable to those of their peers without VI, thereby enhancing their inclusion in motor and sports activities. Findings from the Dutch study indicated that children with VI who participate in organized sports activities demonstrate better OC skills than those who do not engage in such activities. Other studies have highlighted the positive impact of physical activity on the development of gross motor skills in children with VI [43]. Warren [37] emphasized the importance of providing children with VI with ample opportunities to engage in movement experiences, reinforcing the necessity for early motor interventions.

Beyond access to activity opportunities, teacher preparation is a key lever for improving motor outcomes. General physical education (PE) teachers frequently report that teaching students with disabilities—including those with visual impairment—is challenging and that their initial preparation did not equip them with sufficient adapted PE strategies [44,45]. In addition, preservice training specific to visual impairment is often limited, and even experienced educators identify students with VI as among the most difficult populations to teach without targeted guidance [46]. To help address this gap, freely available resources such as the Erasmus+ “MOVE AS YOU ARE” project provide multilingual materials (e.g., a MOOC and a best-practice booklet) that outline practical, evidence-informed adaptations for teaching fundamental movement skills and inclusive sport for children with VI; these resources may support capacity building in schools and communities [47].

In the US and other countries, initiatives such as Camp Abilities have provided opportunities for adapted physical activity in children with VI, with documented positive effects on their motor skills and sports participation [27]. Brian et al. [23] also confirmed the correlation between fundamental motor skills and physical activity in sports camps or daily home environments.

Beyond cross-sectional differences and short-term performance gaps, low motor competence in childhood has been linked to lower physical activity, reduced perceived competence, and adverse developmental trajectories [15,31,48,49]. Moreover, difficulties in motor coordination identified in childhood can persist into adolescence and adulthood, with consequences for education, employment, and social participation [28,29]. Therefore, strengthening access to early, structured motor experiences for Italian children with VI may help to prevent downstream disparities in health and participation.

Therefore, early participation in recreational motor activities and sports among Italian children with VI may serve as a pathway to improve gross motor skills and reduce the gap compared with their peers without VI. With appropriate design and accessibility, these activities can complement rehabilitative interventions. Compared with rehabilitative activities, sports and recreational motor activities offer the advantage of allowing children to experience movement in a playful context alongside their peers, making them more engaging, motivating, and conducive to social interaction. Additionally, unstructured motor activities, such as free play at home, playgrounds, or preschool settings, can play a crucial role in increasing children’s overall motor activity levels [38]. Educating families to be more attentive to the daily movement levels of their children with VI, even during early childhood, could be the key to promote the development of gross motor skills.

### Study Limitations

This study has several limitations. First, the sample was small and nonprobabilistic, which limits generalizability. Second, our international comparisons relied on published aggregate statistics rather than individual-level data. Third, although the TGMD-2 has been validated for use in children with VI, as reported by Houwen [19], objective difficulties were encountered in administering certain tests, particularly OC tasks, to children with blindness. Moreover, more detailed guidelines on how to instruct children with blindness effectively during test administration would be beneficial. Finally, we did not directly measure potentially influential contextual factors across child, family, school, and community levels (e.g., socioeconomic background, home play space, access to organized sport, school PE exposure), which the literature links to differences in fundamental movement skills; these unmeasured variables may partly explain betweenstudy differences and should be addressed in future research.

Although we did not collect individual-level contextual data, some features of the Italian school system may influence movement opportunities. In primary schools, physical education is typically scheduled for about two hours per week, and—following national legislation—since 2022/23, lessons in grade 5 and, since 2023/24, those in grade 4 have been delivered by specialist PE teachers; in the other grades, PE may be taught by generalist teachers. During PE, pupils with disabilities are supported by an individual support teacher, who accesses the role via a postgraduate specialization (TFA Sostegno, 60 ECTS including 300 h of school-based practicum); however, curricula vary by university and do not necessarily include in-depth training in adapted physical education specific to visual impairment. This heterogeneity—together with local policies and resource availability—may influence school-based movement opportunities and limits the generalizability of our results beyond our setting; it also suggests priorities for future research [50,51,52].

Future studies should precisely quantify the daily movement levels of Italian children with VI and determine the extent to which gross motor performance can improve following structured motor activity programs. In addition, examining how poor gross motor performance affects autonomy and social inclusion would provide valuable insights.

## 5. Conclusions

This study was the first to investigate gross motor skill performance in Italian children with VI using the TGMD-2. The findings contribute to the international body of research by providing data on an underrepresented population and highlighting the effect of VI on gross motor competence.

The results confirmed that Italian children with VI exhibited significant delays in LM and OC skills compared to their peers without VI, with a developmental gap of approximately 4–5 years. A comparison with data from other countries suggests that access to structured physical activity and adapted sports programs may positively influence motor outcomes. However, the limited opportunities for children with VI in Italy highlight the need for early interventions and targeted programs to enhance motor skill development.

The data collected in this study provide a foundation for improving early intervention approaches for children with VI in Italy, emphasizing the importance of integrating adapted physical education and structured movement experiences into their daily routines. Future research should explore the long-term effects of motor interventions and examine the influence of different environmental and educational contexts on the motor competence of children with VI.

## Figures and Tables

**Table 1:** Explanation of the modifications applied to the equipment and the original TGMD-2 test procedure.

TGMD-2 Skills	Equipment Modification	Protocol Modification
Run, gallop, hop	Big bright cones to mark start and stop	Examiner at stop line providing vocal feedback for direction.
Leap	Big bright cones to mark start and stop. Three marking disc cones	Examiner at stop line providing vocal feedback for direction. Verbal sign to ‘leap’
Horizontal jump	Rope placed on the floor to mark the start line	Vocal feedback for direction.
Slide	Big bright cones to mark start and stop.	Examiner at stop line providing vocal feedback for direction
Striking a stationary ball	Foam ball with bells placed on a stable stand	
Stationary dribble	Bell basketball	
Catch	Beep ball	Timing mechanism ‘Ready? Catch!’
Kick	Beep ball	Vocal feedback for direction.
Overhand throw	Tennis soundball	Vocal feedback for direction.
Underhand roll	Tennis soundball	Vocal feedback for direction.

**Table 2:** Locomotor and object control scores and total score for children with and without VI. Key result: children with VI showed lower median scores than children without VI—locomotor 25 vs. 43, object control 22 vs. 39, total 49 vs. 82—with *p* < 0.001 for all comparisons.

With vs. Without VI	Children Without VI (*N* = 19)			Children with VI (*N* = 19)			
	M	SD	Mdn	M	SD	Mdn	*p*
Locomotor	42.26	4.34	43.00	25.47	9.69	25.00	<0.001
Object control	36.16	7.22	39.00	22.16	7.94	22.00	<0.001
Total score	78.42	11.08	82.00	47.63	17.15	49.00	<0.001

M, mean; SD, standard deviation; Mdn, median; VI, visual impairment.

**Table 3:** Locomotor and object control scores and total score for children with blindness and with severe VI. Key result: children with blindness scored lower than those with severe VI—locomotor median 22 vs. 32 (*p* = 0.013), object control median 17 vs. 28 (*p* = 0.043), total median 37 vs. 59.5 (*p* < 0.013).

	Blindness (*N* = 9)			Severe VI (*N* = 10)			
	M	SD	Mdn	M	SD	Mdn	*p*
Locomotor	20.00	6.16	22.00	30.40	9.84	32.00	<0.013
Object control	18.11	5.95	17.00	25.80	7.97	28.00	<0.043
Total score	38.11	11.57	37.00	56.20	17.24	59.5	<0.013

M, mean; SD, standard deviation; Mdn, median; VI, visual impairment.

**Table 4:** Locomotor and object control scores and total score for children with severe VI and without VI. Key result: children with severe VI scored lower than children without VI—locomotor median 32 vs. 43, object control median 28 vs. 39, total median 59.5 vs. 82; all *p* < 0.001.

	Without VI (*N* = 19)			Severe VI (*N* = 10)			
	M	SD	Mdn	M	SD	Mdn	*p*
Locomotor	42.26	4.34	43.00	30.40	9.84	32.00	<0.001
Object control	36.16	7.22	39.00	25.80	7.97	28.00	<0.001
Total score	78.42	11.08	82,00	56.20	17.24	59.5	<0.001

M, mean; SD, standard deviation; Mdn, median; VI, visual impairment.

**Table 5:** Locomotor and object control scores and total score for Italian children with severe VI and Dutch children with severe VI. Key result: Italian children with severe VI scored lower than Dutch peers in locomotor (*p* = 0.015) and total score (*p* = 0.02); the object control difference was not significant (*p* = 0.10).

	Italian: Severe VI (*N* = 10)			Dutch: Severe VI (*N* = 7)			
	M	SD	Mdn	M	SD	Mdn	*p*
Locomotor	30.40	9.84	32.00	39.3	2.6	39.0	0.015
Object control	25.80	7.97	28.00	32.7	5.9	31.0	0.10
Total score	56.20	17.24	59.5	72.0	7.7	71.0	0.02

M, mean; SD, standard deviation; Mdn, median; VI, visual impairment.

**Table 6:** Locomotor and object control scores and total score for Italian children with severe VI and US children with B3 vision. Key result: the US B3 group showed higher object control and total scores; the locomotor difference was smaller; see *p*-values in the table (note: B3 is not fully equivalent to WHO-defined severe VI).

	Italian: Severe VI (*N* = 10)			US: B3 VI (*N* = 52)			
	M	SD	Mdn	M	SD	Mdn	*p*
Locomotor	30.40	9.84	32.00	32.87	5.92	-	0.037
Object control	25.80	7.97	28.00	35.90	7.13	-	0.007
Total score	56.20	17.24	59.5	68.77	-	-	

M, mean; SD, standard deviation; Mdn, median; VI, visual impairment.

**Table 7:** Locomotor and object control scores and total score for Italian children with blindness and US children with blindness. Key result: no statistically significant differences were observed for locomotor (*p* = 0.72) or object control (*p* = 0.34).

	Italian: With Blindness (*N* = 9)			US: With Blindness (*N* = 23)			
	M	SD	Mdn	M	SD	Mdn	*p*
Locomotor	20.00	6.16	22.00	-	-	21.0	0.72
Object control	18.11	5.95	17.00	-	-	20.0	0.34
Total score	38.11	11.57	37.00	-	-	-	-

M, mean; SD, standard deviation; Mdn, median.

**Table 8:** Summary table of raw scores obtained by children with VI on the TGMD-2 and corresponding descriptive ratings and age equivalents. Key reading guide: per-child raw scores, age equivalents, and descriptive ratings are shown; for participants >10 years, gross motor quotient/descriptive ratings are not reported (outside the normative range). See Figure 4, Figure 5 and Figure 6 for distributions.

		Locomotor Subtest			Object Control Subtest			Total Score	
Participant	Age	Raw Score	Age Equivalent	Descriptive Ratings	Raw Score	Age Equivalent	Descriptive Ratings	Gross Motor Quotient	Descriptive Ratings
1	12 y, 0 m	30	5 y, 0 m	/	32	6 y, 6 m	/	/	/
2	11 y, 9 m	39	6 y, 6 m	/	34	5 y, 9 m	/	/	/
3	11 y, 9 m	13	<3 y	/	12	<3 y	/	/	/
4	10 y, 11 m	28	4 y, 6 m	Very poor	25	5 y	Very poor	58	Very poor
5	11 y, 3 m	23	3 y, 9 m	/	26	4 y, 6 m	/	/	/
6	11 y, 4 m	25	4 y, 0 m	/	22	4 y, 3 m	/	/	/
7	10 y, 2 m	43	8 y, 6 m	Average	36	7 y, 6 m	Poor	82	Below average
8	9 y, 7 m	9	<3 y	Very poor	14	<3 y	Very poor	46	Very poor
9	9 y, 11 m	34	5 y, 6 m	Very poor	30	5 y, 6 m	Poor	64	Very poor
10	9 y, 6 m	36	6 y, 0 m	Below average	30	6 y, 0 m	Below average	76	Poor
11	9 y, 2 m	33	5 y, 6 m	Poor	22	4 y, 3 m	Very poor	61	Very poor
12	9 y, 1 m	20	3 y, 3 m	Very poor	17	<3 y	Very poor	46	Very poor
13	8 y, 6 m	31	5 y, 0 m	Poor	26	4 y, 6 m	Very poor	61	Very poor
14	7 y, 11 m	25	4 y, 0 m	Very poor	22	3 y, 9 m	Very poor	55	Very poor
15	8 y, 3 m	12	<3 y	Very poor	9	<3 y	Very poor	46	Very poor
16	7 y, 7 m	12	<3 y	Very poor	16	<3 y	Very poor	46	Very poor
17	6 y, 9 m	30	5 y, 0 m	Below average	20	4 y, 0 m	Poor	76	Poor
18	5 y, 2 m	19	3 y, 0 m	Below average	14	<3 y	Poor	70	Poor
19	5 y, 0 m	22	3 y, 6 m	Below average	14	<3 y	Poor	73	Poor

VI, visual impairment; TGMD-2, Test of Gross Motor Development-2.

## Data Availability

The original contributions presented in this study are included in the article. Further inquiries can be directed to the corresponding authors.

## Abbreviations

The following abbreviations are used in this manuscript:

VI	Visual impairment
TGMD-2	Test of Gross Motor Development-2
LC	Locomotor
OC	Object control

## Appendix A

This appendix provides visual examples and brief instructional notes for the VI-appropriate adaptations used during TGMD-2 administration. The set-ups mirror the procedures described in the main manuscript (see Table 1) and were applied consistently across all relevant test items.

### Appendix A.2. Instructional Notes for Explaining Test Items to Children with VI

Because children with blindness cannot learn new skills by visual imitation, task instructions were provided through concise verbal descriptions and brief, task-specific tactile modeling before each trial (e.g., guiding the limb through one correct repetition, or allowing hands to explore key body positions). For practical examples and brief demonstrations of inclusive instruction, see the Erasmus+ MOVE AS YOU ARE MOOC [47] and Lieberman and Haibach [53], pp. 39–40.

## References

[1] Brambring M. (2006). Divergent development of gross motor skills in children who are blind or sighted. J. Vis. Impair. Blind..

[2] Prechtl H.F.R., Cioni G., Einspieler C., Bos A.F., Ferrari F. (2002). Role of vision on early motor development: Lessons from the blind. Dev. Med. Child Neurol..

[3] Hallemans A., Ortibus E., Truijen S., Meire F. (2011). Development of independent locomotion in children with a severe visual impairment. Res. Dev. Disabil..

[4] Elisa F., Josée L., Oreste F.G., Claudia A., Antonella L., Sabrina S., Giovanni L. (2024). Gross motor development and reach on sound as critical tools for the development of the blind child. Brain Dev..

[5] Levtzion-Korach O., Tennenbaum A., Schnitzer R., Ornoy A. (2000). Early motor development of blind children. J. Paediatr. Child Health.

[6] Adelson E., Fraiberg S. (1974). Gross motor development in infants blind from birth. Child Dev..

[7] Bakke H.A., Cavalcante W.A., de Oliveira I.S., Sarinho S.W., Cattuzzo M.T. (2019). Assessment of motor skills in children with visual impairment: A systematic and integrative review. Clin. Med. Insights Pediatr..

[8] Arnaiz Sánchez P. (1994). Deficiencias Visuales y Psicomotricidad: Teoría y Práctica.

[9] Matos M.R. (2006). Análise do Equilíbrio em Postura Ortostática em Crianças com Deficiência Visual por Meio de Parâmetros Estabilométricos. Master’s Thesis.

[10] Matos M., Matos C., Oliveira C. (2010). Equilíbrio estático da criança com baixa visão por meio de parâmetros estabilométricos. Fisioter. Mov..

[11] Lubans D.R., Morgan P.J., Cliff D.P., Barnett L.M., Okely A.D. (2010). Fundamental movement skills in children and adolescents: Review of associated health benefits. Sports Med..

[12] Branta C., Haubenstricker J., Seefeldt V. (1984). Age changes in motor skills during childhood and adolescence. Exerc. Sport Sci. Rev..

[13] Gallahue D.L., Ozmun J.C. (2006). Understanding Motor Development: Infants, Children, Adolescents, Adults.

[14] Gallahue D.L., Donnelly F.C. (2003). Developmental physical education for all children. Palaestra.

[15] Barnett L.M., Lai S.K., Veldman S.L.C., Hardy L.L., Cliff D.P., Morgan P.J., Zask A., Lubans D.R., Shultz S.P., Ridgers N.D. (2016). Correlates of gross motor competence in children and adolescents: A systematic review and meta-analysis. Sports Med..

[16] Houwen S., Hartman E., Visscher C. (2010). The relationship among motor proficiency, physical fitness, and body composition in children with and without visual impairments. Res. Q. Exerc. Sport.

[17] Wagner M.O., Haibach P.S., Lieberman L.J. (2013). Gross motor skill performance in children with and without visual impairments—Research to practice. Res. Dev. Disabil..

[18] Haibach P.S., Wagner M.O., Lieberman L.J. (2014). Determinants of gross motor skill performance in children with visual impairments. Res. Dev. Disabil..

[19] Houwen S., Visscher C., Hartman E., Lemmink K.A.P.M. (2007). Gross motor skills and sports participation of children with visual impairments. Res. Q. Exerc. Sport.

[20] Houwen S., Hartman E., Jonker L., Visscher C. (2010). Reliability and validity of the TGMD-2 in primary-school-age children with visual impairments. Adapt. Phys. Act. Q..

[21] Law M., Stewart D., Pollock N., Letts L., Bosch J., Westmorland M. (1998). Critical Review Form—Quantitative Studies.

[22] Brian A., Starrett A., Pennell A., Haibach-Beach P., Gilbert E., Stribing A., Miedema S.T., Lieberman L. (2021). Longitudinal locomotor competence and body mass index across self-reported gender and vision level for youth with visual impairments: A 3-year investigation. Adapt. Phys. Act. Q..

[23] Brian A., Pennell A., Taunton S., Starrett A., Howard-Shaughnessy C., Goodway J.D., Wadsworth D., Rudisill M., Stodden D. (2019). Motor competence levels and developmental delay in early childhood: A multicenter cross-sectional study conducted in the USA. Sports Med..

[24] Brian A., Starrett A., Haibach-Beach P., De Meester A., Taunton Miedema S., Pennell A., Lieberman L.J. (2022). Perceived motor competence mediates the relationship between gross motor skills and physical activity in youth with visual impairments. Res. Q. Exerc. Sport.

[25] Stribing A., Pennell A., Gilbert E.N., Lieberman L.J., Brian A. (2022). Self-perceptions, parents’ perceptions, metaperceptions, and locomotor skills in adolescents with visual impairments: A preliminary investigation. J. Mot. Learn. Dev..

[26] Brian A., Fisher J.R., Miedema S.T., Pennell A., Lieberman L.J. (2021). The initial psychometric properties for the Total Body Developmental Sequences for youth with visual impairments. J. Dev. Phys. Disabil..

[27] Caron V., Allegranza L., Lieberman L., Haibach-Beach P. (2024). Camp Abilities—An educational sports camp for children and youth with visual impairment: A systematic review. Br. J. Vis. Impair..

[28] Cantell M.H., Smyth M.M., Ahonen T.P. (1994). Clumsiness in adolescence: Educational, motor, and social outcomes of motor delay detected at 5 years. Adapt. Phys. Act. Q..

[29] Cousins M., Smyth M.M. (2003). Developmental coordination impairments in adulthood. Hum. Mov. Sci..

[30] Winnick J.P. (1985). The performance of visually impaired youngsters in physical education activities: Implications for mainstreaming. Adapt. Phys. Act. Q..

[31] Brian A., Getchell N., True L., De Meester A., Stodden D.F. (2020). Reconceptualizing and operationalizing Seefeldt’s proficiency barrier: Applications and future directions. Sports Med..

[32] Houwen S., Visscher C., Lemmink K.A.P.M., Hartman E. (2009). Motor skill performance of children and adolescents with visual impairments: A review. Except. Child.

[33] Aki E., Atasavun S., Turan A., Kayihan H. (2007). Training motor skills of children with low vision. Percept. Mot. Skills.

[34] Fazzi E., Signorini S.G., Bova S.M., Ondei P., Bianchi P.E. (2005). Early intervention in visually impaired children. Int. Congr. Ser..

[35] Jazi S.D., Purrajabi F., Movahedi A., Jalali S. (2012). Effect of selected balance exercises on the dynamic balance of children with visual impairments. J. Vis. Impair. Blind..

[36] Santos L., Passos J., Rezende A. (2007). Os efeitos da aprendizagem psicomotora no controle das atividades de locomoção sobre obstáculos em crianças com deficiência da visão. Rev. Bras. Educ. Espec..

[37] Warren D.H. (1994). Blindness and Children: An Individual Differences Approach.

[38] Schneekloth L.H. (1989). Play environments for visually impaired children. J. Vis. Impair. Blind..

[39] Ulrich D.A. (2000). Test of Gross Motor Development.

[40] Ulrich D.A. (2017). Introduction to the special section: Evaluation of the psychometric properties of the TGMD-3. J. Mot. Learn. Dev..

[41] Brian A., Taunton S., Lieberman L.J., Haibach-Beach P., Foley J., Santarossa S. (2018). Psychometric properties of the Test of Gross Motor Development-3 for children with visual impairments. Adapt. Phys. Act. Q..

[42] Murphy F.M., O’Driscoll M. (1989). Observations on the motor development of visually impaired children: Interpretations from video recordings. Physiotherapy.

[43] Okely A.D., Booth M.L., Patterson J.W. (2001). Relationship of physical activity to fundamental movement skills among adolescents. Med. Sci. Sports Exerc..

[44] Lirgg C.D., Gorman D.R., Merrie M.D., Shewmake C. (2017). Exploring Challenges in Teaching Physical Education to Students with Disabilities. Palaestra.

[45] Wilson K., Dieringer S., Klay K., McPherson A. (2022). A Descriptive Probe into Current Introduction to Adapted Physical Education Courses Across the United States. Phys. Educ..

[46] Lieberman L.J., Bryant L., Brown D., McHugh L., Robinson B. (2022). A Qualitative Inquiry of a Three-Month Virtual Practicum for Preservice Teachers of Students with Visual Impairments. Int. J. Environ. Res. Public Health.

[47] Move As You Are Consortium MOVE AS YOU ARE—Resources: Booklet on Best Practices and MOOC for Including Children with Visual Impairments in Sport. https://www.moveasyouare.eu/resources/.

[48] Cattuzzo M.T., Dos Santos Henrique R., Ré A.H.N., de Oliveira I.S., de Sousa Moura M., Melo B.M., de Araújo R.C., Stodden D. (2016). Motor competence and health related physical fitness in youth: A systematic review. J. Sci. Med. Sport.

[49] Robinson L.E., Stodden D.F., Barnett L.M., Lopes V.P., Logan S.W., Rodrigues L.P., D’Hondt E. (2015). Motor Competence and its Effect on Positive Developmental Trajectories of Health. Sports Med..

[50] He Y., Zhou L., Liang W., Liu Q., Liu W., Wang S. (2024). Individual, family, and environmental correlates of fundamental motor skills among school-aged children: A cross-sectional study in China. BMC Public Health.

[51] Ministero Dell’istruzione e del Merito Nota 12 Aprile 2023, Prot. 26952—Dotazioni Organiche del Personale Docente a.s. 2023/2024 (Richiamo Alla Nota 2116/2022 e Indicazioni su Classi Quarte e Quinte Per Educazione Motoria). https://www.edscuola.eu/wordpress/wp-content/uploads/2023/09/Nota-12-aprile-2023-AOODGPER-26952.pdf.

[52] Ministero Dell’istruzione, Dell’università e Della Ricerca Decreto Ministeriale 10 Settembre 2010, n. 249—Regolamento Sulla Formazione Iniziale Dei Docenti (Art. 13: Percorsi di Specializzazione Per il Sostegno Didattico—60 CFU, 300 Ore Di Tirocinio). https://www.mim.gov.it/documents/20182/0/2010%2B09%2B10_DM249_2010.pdf.

[53] Lieberman L.J., Haibach P. (2016). Gross Motor Development Curriculum for Children with Visual Impairments.

